# Circ_0027599/PHDLA1 suppresses gastric cancer progression by sponging miR-101-3p.1

**DOI:** 10.1186/s13578-018-0252-0

**Published:** 2018-11-06

**Authors:** Liang Wang, Jingyan Shen, Yushan Jiang

**Affiliations:** Department of Gastroenterology, Shanghai Jinshan Branch of the Sixth People’s Hospital, 147 Health Road, Zhujing Town, Jinshan District, Shanghai, 201500 China

**Keywords:** Circ_0027599, miR-101-3p.1, Gastric cancer, PHLDA1

## Abstract

**Background:**

Pleckstrin homology-like domain family A member 1 (PHLDA1) is a tumor suppressor gene in gastric cancer, but its role regulated by circular RNAs (circRNAs) is not known. CircRNAs are important regulators in cancer growth and progression, however, the molecular roles of circRNAs in gastric cancer are rarely known. The study was aimed to investigate the role of circRNAs in regulating PHLDA1 expression in gastric cancer.

**Results:**

The circRNA expression profile in the gastric cancer tissues by circRNA microarray showed that hsa_circ_0027599 (circ_0027599) was significantly down-regulated in gastric cancer patients and cells when comparing with the controls. Circ_0027599 overexpression suppressed gastric cancer cell proliferation and metastasis. By using bioinformatics tools and luciferase reporter assays, circ_0027599 was verified as a sponge of miR-101-3p.1 (miR-101) and suppressed cancer cell survival and metastasis. It was also verified that PHLDA1 was regulated by circ_0027599 in gastric cancer cells.

**Conclusions:**

The study uncovered that PHLDA1 was regulated by circ_0027599/miR-101, which suppressed gastric cancer survival and metastasis in gastric cancer.

## Background

Gastric cancer is a common type of cancer from digestive system in the world and there are near one million new gastric cancer cases every year [1, 2]. There are great achievements in gastric cancer therapy and diagnosis, but the prognosis of gastric cancer is still poor and the 5-year survival rate of gastric cancer is below 30% [2]. Therefore, it is pivotal to identify or find new biomarkers and therapeutic targets for improving gastric cancer prognosis. Better elucidating the mechanisms of gastric tumorigenesis and aggressiveness is important for improving the therapeutic efficiency of gastric cancer [2].

Pleckstrin homology-like domain family A member 1 (PHLDA1) protein is encoded by the *PHLDA1* gene [3]. This gene encodes an evolutionarily conserved proline-histidine rich nuclear protein [3]. PHLDA1 could function as an oncogene or a tumor suppressor gene in cancers. In oral cancer, PHLDA1 was overexpressed, which acted as an apoptosis suppressor and was associated with advanced clinical stage of oral cancer [4]. However, in oral squamous cell carcinoma, the expression of PHLDA1 was very low and acted as a tumor suppressor [5]. In colon cancer, PHLDA1 was a putative epithelial stem cell marker in the human small and large intestine and contributes to cell migration and proliferation [6]. In estrogen receptor (ER) positive breast cancer, ER and NF-κB worked together to upregulate PHLDA1 expression directly through enhanced transcription and indirectly through repression of miR-181a and miR-181b [7]. Down-regulation of PHLDA1 protein was found in breast cancer and patients with low PHLDA1 protein levels had shorter disease-free survival and overall survival than patients with high PHLDA1 protein levels, which suggested that it was a strong predictor of poor prognosis for breast cancer patients [8–10]. PHLDA1 may be down-regulated in breast cancer with ER negative [11]. In gastric cancer, PHLDA1 is down-regulated and may be a tumor inhibitor [12]. So, PHLDA1 acts as an oncogene or a suppressor in tumor depending on their background.

Circular RNAs (circRNAs) are a type of non-coding RNA molecules, shape a covalently closed continuous loop without 5′-3′ polarity and polyA tail and play a significant role in gene expression through competitive binding miRNA without protein translation capacity [13–15]. Increasing evidences confirm that circRNA is implicated in the progression and development of various cancer through sponging miRNA to affect targeted gene [13–15]. In this study we attempted to characterize the molecular mechanisms of the suppressing role of PHLDA1 in gastric cancer. Circ_0027599 was a potential circRNA that regulated PHLDA1 expression. Further investigation was carried out to explore the role of circ_0027599 in gastric cancer cell survival and metastasis. The molecular mechanism of circ_0027599 was also studied. It was found that circ_0027599 sponging with miR-101 functions as a tumor suppressor in gastric cancer.

## Methods

### Gastric cancer samples

Gastric cancer samples and their matched normal adjacent tissues were gained from gastric cancer patients with median age 56.5 years at Shanghai Jinshan Branch of the Sixth People’s Hospital (Shanghai, China). The diagnosis was based on pathological evidence. The samples of the gastric cancer patients were collected and stored at − 80 °C. All samples were collected after the patients provided written informed consent from the Ethics Boards of Shanghai Jinshan Branch of the Sixth People’s Hospital.

### Cell culture

All the cell lines including gastric cancer cell lines (SGC-7901, MGC-803, HGC-27, MKN-45, MKN-28), and human stomach epithelial HPSEC cells used in the study were primarily obtained from American Type Culture Collection (Rockville, MD, USA). The cells were cultured at 37 °C with 5% CO_2_ according to the standard protocols, with DMEM-F12 containing 10% fetal bovine serum, penicillin (100 U/ml) and streptomycin (100 µg/ml).

### CircRNA, miRNA, siRNA or plasmid transfection

MiR-101 mimics (miR-101) and its negative control (miR-control), a miR-101 inhibitor and its control were ordered from the Liyang Biotech (Wuhan, China). circ_0027599, or circ_0027599 siRNAs were ordered from the Liyang Biotech (Wuhan, China). PHLDA1 siRNAs and its control were obtained from RiboBio (Guangzhou, China). Lipofectamine 2000 (Invitrogen) was used for miRNAs or siRNA transfection.

### Colony formation assay

Gastric cancer cells were seeded in 6-well plates. Cells were transfected with circ_0027599 siRNA, miR-101 or PHDLA1 siRNAs and cultured in the normal condition. The cells were cultured for 10 days, washed with 1× PBS, fixed with 70% ethanol for 5 min and stained with 0.5% crystal violet for 3 min at room temperature. The colonies (> 50 cells) were counted. All experiments were performed at least three times.

### Cell proliferation

Gastric cancer cells were seeded in 6-well plates and transfected with circ_0027599, miR-101 or miR-101 or PHLDA1 siRNAs and cultured in the normal condition. Cell survival ability was tested by CCK8 (Meilunbio, Shanghai, China) assay.

### Dual luciferase reporter assay

PHLDA1 promoter activity was examined using Dual-Luciferase Reporter Assay System (Promega) according to the manufacturer’s instructions. Cells were seeded in 24-well plates and transfected the PHLDA1 3′UTR, wild type or mutant reporter constructs and Renilla plasmid by using lipofectamine 2000 (Invitrogen). Luciferase activity was performed after transfection for 48 h using the Dual luciferase assay system (Promega, WI).

### Migration and invasion assay

Cell migration was determined using transwell system (Corning, NY). Gastric cancer cells (1.0 × 10^4^) were transfected with circ_0027599, miR-101 or PHLDA1 siRNAs for 24 h, and then seeded in the upper chamber and allowed to migrate or invade toward the chamber. After 12 h (for migration assays without matrigel coating) or 24 h (for invasion assays with matrigel coating), the migrated or invaded cells underside of the membrane were counted and the relative migration or invasion was analyzed.

### RNA extraction and real-time PCR analysis

Gastric cancer cells were transfected with circ_0027599, miR-101 or PHLDA1 siRNAs or the controls for 48 h and then total RNA was isolated for real time RT-PCR analysis. The expression level of miRNAs was defined based on the threshold cycle (Ct), and relative expression levels were calculated using the 2^−ΔΔCt^ method, using the expression level of the U6 snRNA as a reference gene.

### Western blotting

Cultured cells were harvested and lysed with RIPA buffer containing the protease inhibitors on ice for 30 min. Protein was separated by SDS-PAGE, transferred onto nitrocellulose membrane and probed with primary antibodies including PHLDA1 or GAPDH and then horseradish peroxidase—labeled secondary antibodies. The protein band signals were visualized using an ECL.

### Statistical analysis

All data were analyzed using the SPSS 18.0 (SPSS, Chicago, IL, USA) or Excel. Every experiment was completed independently at least three times. A p value < 0.05 was considered significant.

## Results

### PHLDA1 decreases gastric cancer cell survival and metastasis

**Fig. 1:**
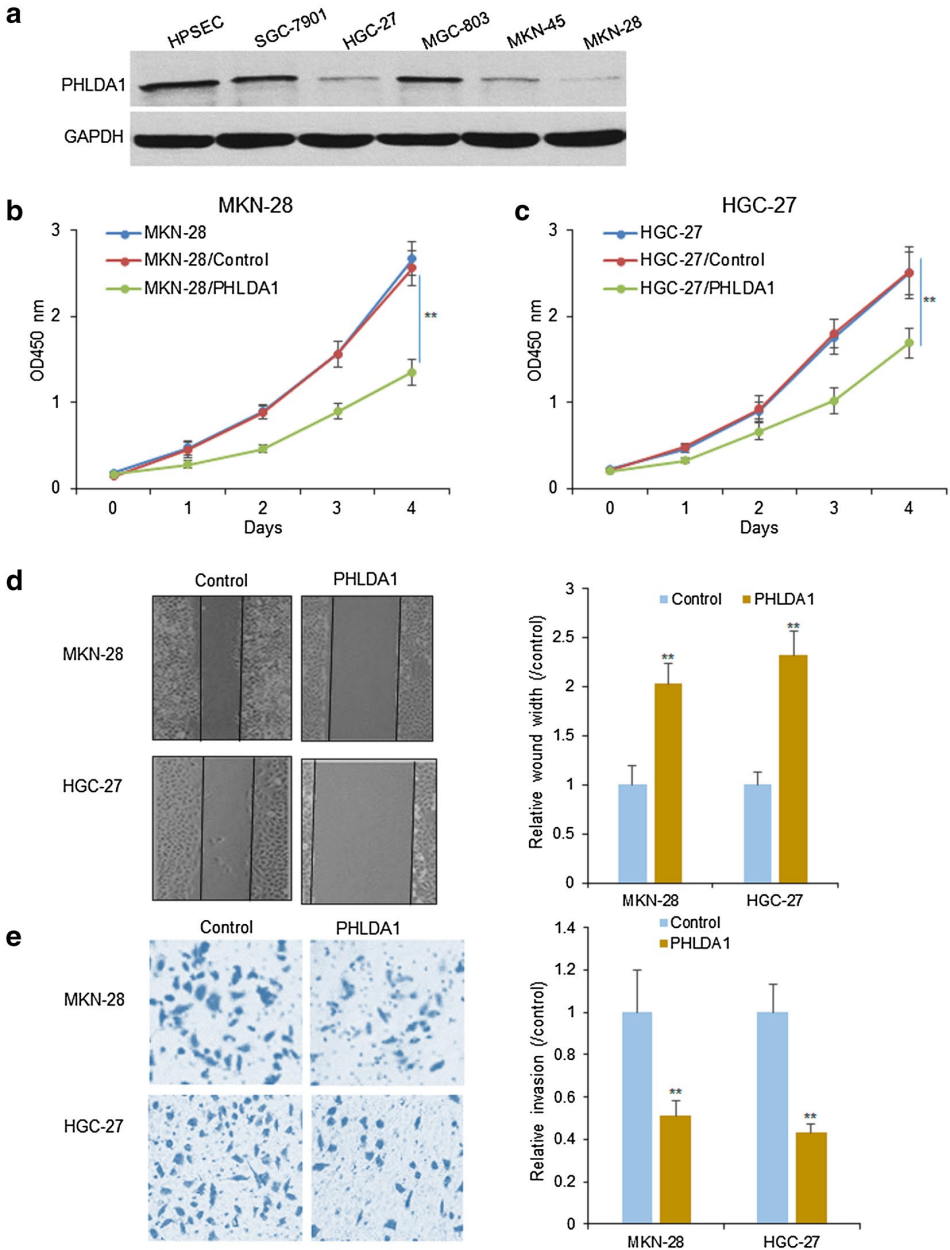
PHLDA1 decreases gastric cancer cell survival and metastasis. **a** PHLDA1 protein levels in gastric cancer cell lines. PHLDA1 protein was examined using western blotting. **b**, **c** PHLDA1 inhibited gastric cancer cell proliferation. MKN-28 and HGC-27 cells were transfected with PHLDA1 for 24 h. Cells were collected and seeded in 96 well plates for CCK8 assay. **d** PHLDA1 inhibited gastric cancer cell migration. MKN-28 and HGC-27 cells were transfected with PHLDA1 for 24 h. Cells were collected and seeded in 12-well plates for wound healing assay. **e** PHLDA1 inhibited gastric cancer cell invasion. MKN-28 and HGC-27 cells were transfected with PHLDA1 24 h. Cells were collected and seeded in the up-chamber of transwell system coated with Matrigel. **p < 0.01

To investigate the role of PHLDA1 on gastric cancer cell survival ability, firstly, SGC-7901, MGC-803, HGC-27, MKN-45, MKN-28 gastric cancer cells and human stomach epithelial cells (HPSEC) were used to examine PHLDA1 expression, it was found that PHLDA1 expression was lower in MKN-28 and HGC-27 cell lines than the other cell lines (Fig. 1a). MKN-28 and HGC-27 cell lines were selected to the following experiments. MKN-28 and HGC-27 cells were transfected with PHLDA1 and cell proliferation was measured by CCK8 method. We found that MKN-28 and HGC-27 cells grew lower than the cells with the control (Fig. 1b, c). We also found the migration ability decreased greatly in the MKN-28 and HGC-27 cells with PHLDA1 overexpression (Fig. 1d). Transwell system was used to observe the gastric cancer cell invasion ability and the result showed that the invaded cells became less in MKN-28 and HGC-27 cells with PHLDA1 overexpression (Fig. 1e).

### PHLDA1 is a potential target gene of miR-101

**Fig. 2:**
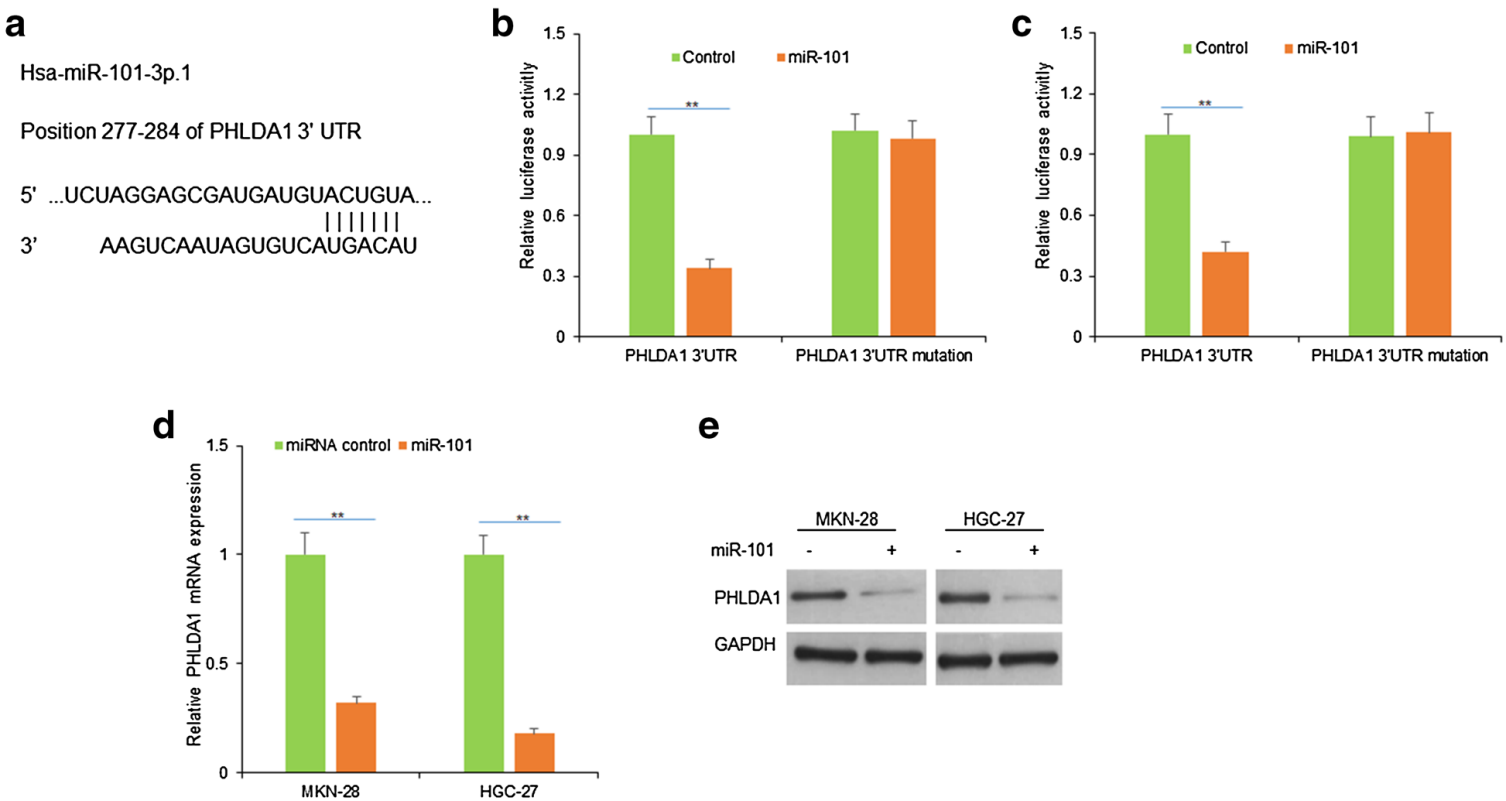
PHLDA1 is regulated by miR-101 in gastric cancer cells. **a** PHLDA1 was a predicted as a target gene of miR-101 by TargetScan. **b**, **c** Luciferase assay was used to evaluate PHLDA1 3′UTR (wide typed or mutated one) activity in MKN-28 and HGC-27 cells respectively. **d** miR-101 decreased PHLDA1 mRNA expression in gastric cancer cells. MKN-28 and HGC-27 cells were transfected with miR-101 and then its expression was detected by qRT-PCR. **e** miR-101 decreased PHLDA1 protein expression in gastric cancer cells. MKN-28 and HGC-27 cells were transfected with miR-101 and then PHLDA1 protein level was examined by western blotting. **p < 0.01

It was predicted that PHLDA1 was the target gene of miR-101-3p.1 (miR-101) by TargetScan (Fig. 2a). The luciferase assay was used to test whether PHLDA1 was the target gene of miR-101, and the results showed that luciferase activity in MKN-28 and HGC-27 cells was down-regulated significantly when the cells were co-transfected with miR-101 mimics and wide type of PHLDA1 3′UTR (Fig. 2b, c). To know whether miR-101 regulates PHLDA1 expression in gastric cancer cells, MKN-28 and HGC-27 cells were transfected with miR-101 and miR-101 was effectively up-regulated (Fig. 2d). We used RT-PCR to verify the most potential target genes and found that PHLDA1 mRNA was significantly down-regulated in gastric cancer cells with miR-101 (Fig. 2e). The result was confirmed by western blotting (Fig. 2e).

### Circ_0027599 acted as a sponge of miR-101 in gastric cancer

**Fig. 3:**
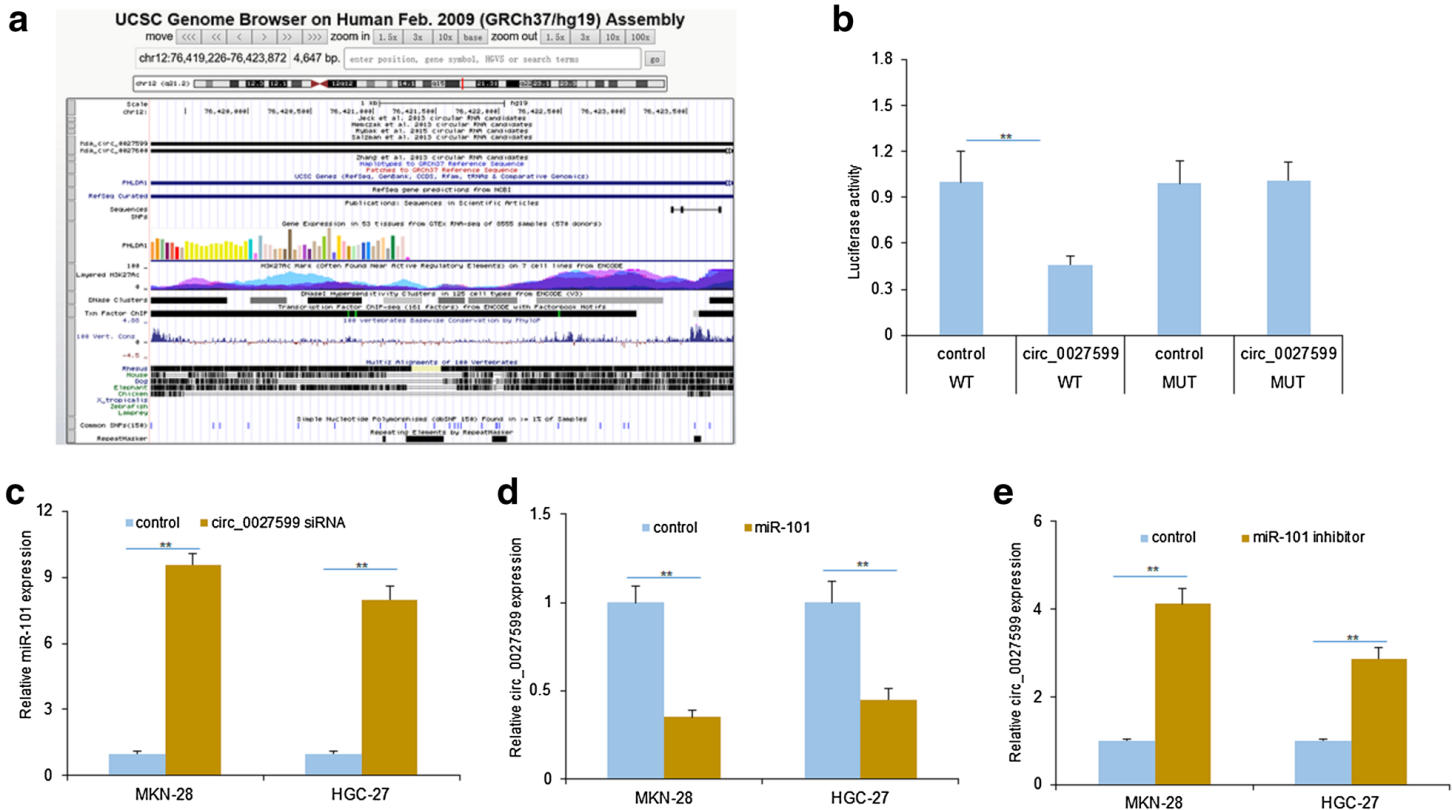
Circ_0027599 acted as a sponge of miR-101 and up-regulates PHLDA1 expression in gastric cancer. **a** The information of circ_0027599 from circRNA database. **b** The luciferase activity decreased in MKN-28 cells transfected with miR-101. The luciferase activity was assayed by dual luciferase reporter system. **c** miR-101 levels were up-regulated in MKN-28 and HGC-27 cells transfected with circ_0027599 siRNA. MiR-101 levels were analyzed by qRT-PCR. **d** Circ_0027599 expression was down-regulated in MKN-28 and HGC-27 cells transfected with miR-101 mimics. Circ_0027599 levels were analyzed by qRT-PCR. **e** Circ_0027599 expression was up-regulated in MKN-28 and HGC-27 cells were transfected with miR-101 inhibitors. Circ_0027599 levels were analyzed by qRT-PCR. **p < 0.01

For further exploring the molecular role of PHLDA1 regulated by miR-101, PHLDA1 may be regulated by circ_0027599 (Fig. 3a). Circ_0027599 locates in chromosome 12 and similar to the other circRNAs, lack of a poly A tail, resistance to RNase R, and longer half-life (they were confirmed by other researcher in our lab and the data will be submitted in the further). To know whether circ_0027599 regulates miR-101, luciferase reporter assay was used to confirm whether circ_0027599 combined with miR-101, and the luciferase activity decreased in MKN-28 and HGC-27 cells which were transfected with miR-101 (Fig. 3b). To know the regulation of circ_0027599 on miR-101 expression, MKN-28 and HGC-27 cells were transfected with circ_0027599 siRNA and the result indicated that miR-101 expression was up-regulated in cells (Fig. 3c). MKN-28 and HGC-27 cells were transfected with miR-101 inhibitors, and circ_0027599 expression was up-regulated (Fig. 3d). The molecular characteristics of circ_0027599, should be confirmed including its lack of a poly (A) tail, resistance to RNase R, and longer half-life.

### Circ_0027599 suppressed gastric cancer cell proliferation and metastasis by miR-101/PHLDA1 expression

**Fig. 4:**
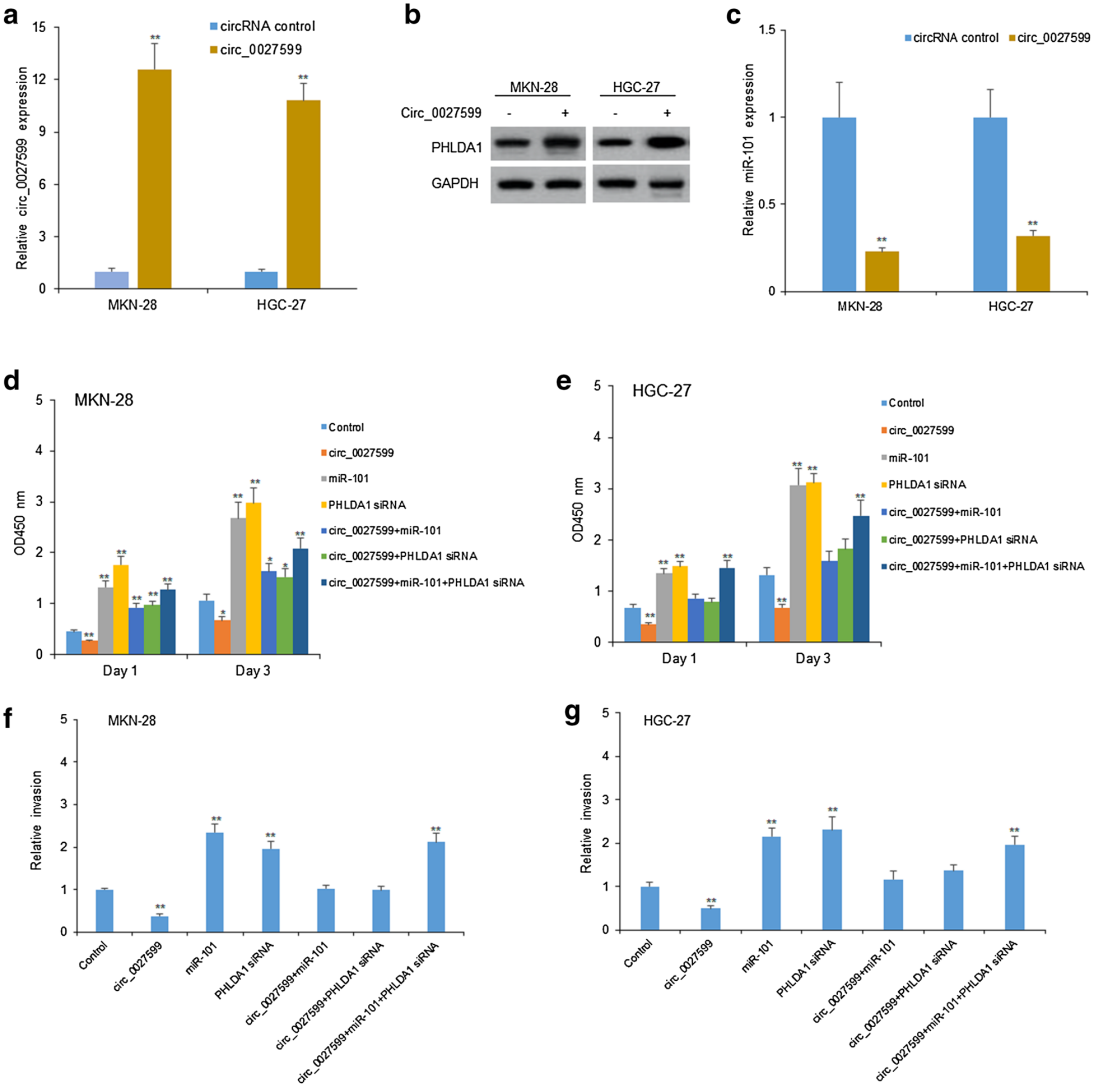
Circ_0027599 suppressed gastric cancer cell proliferation. **a** Circ_0027599 expression in MKN-28 and HGC-27 cells. MKN-28 and HGC-27 cells were transfected with circ_0027599 and circ_0027599 levels were analyzed by qRT-PCR. **b** PHLDA1 levels in MKN-28 and HGC-27 cells. MKN-28 and HGC-27 cells were transfected with circ_0027599 and PHLDA1 levels were analyzed by western blotting. **c** miR-101 levels in MKN-28 and HGC-27 cells. MKN-28 and HGC-27 cells were transfected with circ_0027599 and miR-101 levels were analyzed by western qRT-PCR. **d**, **e** Circ_0027599 reduced gastric cancer cell proliferation. MKN-28 and HGC-27 cells were transfected with circ_0027599, miR-101, or PHLDA1 siRNA and survival ability was analyzed by CCK8 assay at the day 1 and day 3. **f**, **g** Circ_0027599 reduced gastric cancer cell invasion. MKN-28 and HGC-27 cells were transfected with circ_0027599, miR-101, or PHLDA1 siRNA and invasion ability was analyzed by transwell chambers with matrigel treatment. **p < 0.01; *p < 0.05

To investigate the role of circ_0027599 in gastric cancer cell growth, MKN-28 and HGC-27 cells were transfected with circ_0027599 using lentivirus vectors. The results indicated that circ_0027599 was effectively up-regulated in the gastric cancer cells (Fig. 4a), PHLDA1 protein was up-regulated (Fig. 4b) and miR-101 was down-regulated (Fig. 4c). Then, the cellular functions were assayed. MKN-28 and HGC-27 cells were transfected with circ_0027599, miR-101 or PHLDA1 siRNAs, and cell survival and metastasis ability were evaluated. CCK8 assay showed that circ_0027599 decreased the colony numbers in the gastric cancer cells with miR-101 overexpression or down-regulating PHLDA1 expression (Fig. 4d, e). Transwell chamber with matrixgel treatment to analyze the invasion of gastric cancer cells. The data indicated that circ_0027599 decreased the invasion or migration ability of the gastric cancer cells with PHLDA1 siRNA or miR-101 transfection (Fig. 4f, g). The results showed that the circ_0027599 decreased the proliferation and metastasis of gastric cancer cells via miR-101/PHLDA1.

### Circ_0027599 was down-regulated, positively related to PHLDA1 and negatively related to miR-101 in gastric cancer tissues

**Fig. 5:**
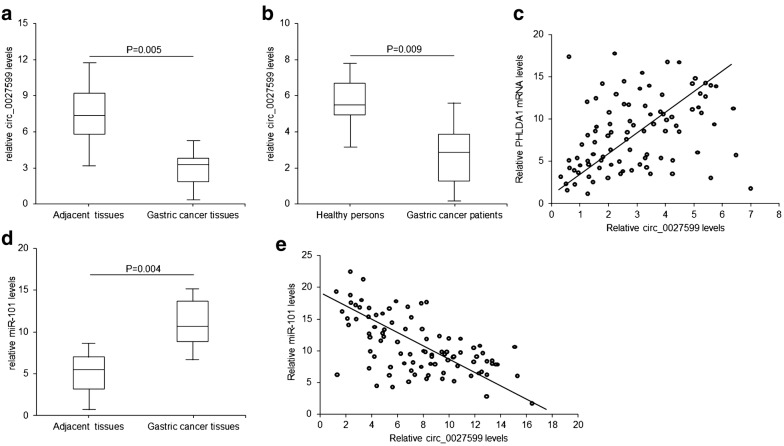
Circ_0027599 was down-regulated in gastric cancer tissues and cell lines. **a** Circ_0027599 was down-regulated in the gastric cancer tissues samples. Circ_0027599 expression was detected using qRT-PCR. **b** Circ_0027599 was down-regulated in the blood samples from gastric cancer patients. Circ_0027599 expression was detected using qRT-PCR. **c** The relationship between circ_0027599 and PHLDA1 levels in gastric cancer tissues. **d** MiR-101 was up-regulated in gastric tissues. **e** The relationship between circ_0027599 and miR-101 levels in gastric cancer tissues

To verify circ_0027599 expression in samples of the gastric cancer, 78 cases were collected for measuring circ_0027599 expression by real time RT-PCR. The data showed that the average level of circ_0027599 was lower in 78 pairs of cancer tissues than it in adjacent normal tissues (Fig. 5a). In gastric cancer cells, circ_0027599 was also lower in the blood samples of gastric cancer than it in the healthy persons (Fig. 5b). To learn the relationship of circ_0027599 levels with clinic features of gastric cancer. The analyzed results showed that circ_0027599 was not related the ages of gastric cancer patients (Table 1). There was a negative relationship between circ_0027599 expression with tumor size, depth of invasion, lymph node distant metastasis, distant metastasis and TNM staging (Table 1). We also analyzed the relationship between circ_0027599 and PHLDA1 levels in gastric cancer tissues, and the data indicated that circ_0027599 expression was positively associated with PHLDA1 expression (Fig. 5c). MiR-101 was up-regulated in gastric tissues (Fig. 5d). The relationship between circ_0027599 and miR-101 levels in gastric cancer tissues indicated that circ_0027599 expression was positively associated with PHLDA1 expression (Fig. 5e).

**Table 1:** The relationship between circ_0027599 and clinic features in gastric cancer

Clinicopathological variables	Cases/group	Circ_0027599		p value
		Low	High	
Age (years)				
< 60	31	22	9	0.132
≥ 60	47	29	18	
Sex				
Male	36	19	17	0.865
Female	42	22	20	
Tumor size (cm)				
< 5	43	29	14	0.035
≥ 5	35	23	12	
Histological type				
Diffuse	36	27	11	0.041
Intestinal	42	25	17	
Depth of invasion				
T1 + T2	31	20	11	0.024
T3 + T4	47	29	18	
Lymph node distant metastasis				
N0 + N1	28	18	10	0.019
N2 + N3	50	36	14	
Distant metastasis				
M0	32	22	10	0.011
M1	46	33	13	
TNM				
I + II	27	18	9	0.012
III + IV	51	36	15	

## Discussion

In the present work, we observed that PHLDA1 expression was significantly down-regulated in gastric cancer cells and tissues. We demonstrated that PHLDA1 suppressed the migratory and invasive abilities of MKN-28 and HGC-27 gastric cancer cells. Moreover, Bioinformatics analysis and luciferase activity assay demonstrated that PHLDA1 was a direct target gene of miR-101. The miR-101 mimics can significantly decrease protein expression level of PHLDA1 in the gastric cancer cells. It was also showed that circ_0027599 played as a tumor suppressor and regulated gastric cancer cell proliferation and metastasis via down-regulating miR-101.

CircRNAs are potential diagnosis markers in tumor. Due to the stability of circular structure, circRNAs are influenced by the digestion of RNA enzyme, which makes circRNAs enrichment in body fluid including blood. It is reported that there are so many circRNAs found in cancer and the significant circRNAs are screened by circRNA array or next generating sequencing [16, 17]. For example, it was found that 357 circRNAs in lung cancer tissues were dysregulated by circRNA array and there were five significant circRNAs identified by qRT-PCR [17]. It was found that 19 circRNAs were downregulated and 8 were upregulated in breast cancer tissues by circRNA array and there were significant circRNAs identified by qRT-PCR [18]. Liu et al. found that some significant circRNAs were discovered in osteosarcoma by circRNA array and 12 circRNAs were confirmed in both osteosarcoma cell lines and tissues [19]. There are many reported circRNAs in gastric cancer. Human circ_0074362 and circ_0003159 expression was downregulated in gastric cancer, which suggested their association with clinical features and potential diagnostic values [16, 17]. It was also reported that circ_100269 was downregulated and suppressed tumor cell growth by targeting miR-630 in gastric cancer [18]. Decreased expression of hsa_circ_0001895 in human gastric cancer and its clinical significances [19]. These examples showed that down-regulated circRNAs in gastric cancer. There are reports that some up-regulated circRNAs in gastric cancer. For instance, circ_0000190 [20], circPVT1 [21], circ_0047905 [22], circ_0138960 [22], circ_0047905 [22], circ_0138960 [22], circRNA7690-15 [22], circ_0000520 [23], circ_0023642 [24] and others [25]. The up-regulated circRNAs functioned as a potential marker, a prognostic marker, a proliferative marker [20–24]. In this study, circ_0027599 expression was validated to be significantly down-regulated in 78 pairs of gastric cancer tissue comparing to the adjacent normal tissue. It suggested that circ_0027599 was a potential marker which will benefit for gastric cancer diagnosis and therapy.

Bioinformatics analysis, an emerging discipline, was useful for biologist to explore the underlying mechanism and interrelation of molecules. As we all known, circRNA can sponge mic-RNAs to regulate their targeted genes. So, we predicted miRNA sponged by circ_0027599 using bioinformatics analysis and the result demonstrated that miR-101 was sponged with circ_0027599. Dual luciferase reporter assay and real-time fluorescence quantitative PCR experiments confirmed that miR-101 was negatively regulated by circ_0027599 in gastric cancer cells. Further bioinformatics analysis revealed the mapping of pathways in cancer mediated by miR-101. In our present study, functional analysis revealed that miR-101 stimulated cell growth, migration, and invasion, indicating that miR-101 could function as an onco-miRNA in gastric cancer by targeting PHLDA1. The biological processes and molecular mechanisms underlying miR-101 still remain unclear in gastric cancer, and further functional studies are warranted to address these unsolved issues.

Taken together, the study demonstrated that there were significant differences in the circRNAs expression profiles from gastric cancer tissue and their adjacent normal tissues. After identification by real time RT-PCR, it was found that circ_0027599 was down-regulated in gastric cancer and suppressed cell proliferation and metastasis. The clinical data indicated that circ_0027599 was a potential diagnosis marker of gastric cancer. It was also verified that miR-101 bond to circ_0027599. The further molecular mechanism needs for further investigation.

## Conclusions

The study uncovered that PHLDA1 was regulated by circ_0027599/miR-101, which suppressed gastric cancer survival and metastasis in gastric cancer.
